# Efficiency of the leaves and fruits of *Aegle marmelos* methanol extract (L.) Correa and their relative hepatotoxicity induced by CCL_4_ and identification of their active constituents by using LC/MS/MS

**DOI:** 10.1016/j.toxrep.2018.09.005

**Published:** 2018-10-13

**Authors:** Nabaweya A. Ibrahim, Magdy M.D. Mohammed, Hanan F. Aly, Sanaa Ahmed Ali, Doaa-Abd Al-Hady

**Affiliations:** a Pharmacognosy Department, National Research Centre (NRC), Dokki, 12622, Giza, Egypt; b Therapeutic Chemistry Department, National Research Centre (NRC), 33 EL Bohouth St. (former EL Tahrirst.), Dokki, Giza, 12622, Egypt; c Institut für Umweltforschung (INFU), Technische Universität Dortmund, Otto-Hahn-Strasse 6, D-44221 Dortmund, Germany

**Keywords:** *A. marmelos*, LC/MS*/*MS, Alkaloids, Flavonoids, CCl_4_, Hepatotoxicity, Liver function, Histopathology

## Abstract

The methanol extracts of both leaves and fruits (MEL & MEF) of *A. marmelos* (L.) Correa (family Rutaceae) were analyzed by using analytical method based on liquid chromatography-tandem mass spectrometry (LC/MS/MS). The objective of this study was to identify the active constituents of (MEL & MEF) of *A. marmelos*. Six, alkaloids namely aeglemarmelosine, marmesiline aegelinoside, shahidine, anhydromarmeline and *N*-2-methoxy-2-(4-methoxyphenyl) ethylcinnamide and two flavonoids, rutin and kaempferol-3-*O*-rutinoside in leaves were identified. Two alkaloids marmesiline and shahidine in the methanol extracts of fruits, also have been identified. Moreover, the efficiency of extracts was performed for measuring the reducing hepatotoxicity effect induced by carbon tetrachloride (CCl_4_) in mice. Accordingly, several biochemical parameters were performed such as lipid profile in serum, liver functions enzyme activities, glycolytic enzyme activities. In addition, LDH and SDH were investigated. The results obtained demonstrated, significant increase in lipid profile, liver function biomarkers in addition to glycolytic enzyme activities in CCl_4_-induced hepatotoxicity. Histopathological examination confirmed the biochemical results. Treatment of intoxicated mice with (MEL & MEF) of *A. marmelos* showed amelioration signs in biochemical findings as well as at cellular level. It could be concluded that both MEL & MEF can be used clinically for their potential effect as a hepatoprotective that normalized liver function biomarkers, hepatic architecture and restore physiologically status of the body against CCl_4_ intoxication.

## Highlights

• Carbon tetrachloride toxicity on male mice was studied.
• Leaves and fruits of *Aegle marmelos* methanol extracts (L.) Correa and their relative hepatotoxicity were analyzed.
• Flavonoids; Marmesiline and Shahidine were identified from the MeOH ext.of both leaves and fruits.
• The potency *Aegle marmelos* will open new areas for the development of safe and cheap hepatoprotective drugs from natural sources for treatment of a wide range of liver diseases.

## Graphical abstract

**Figure:**
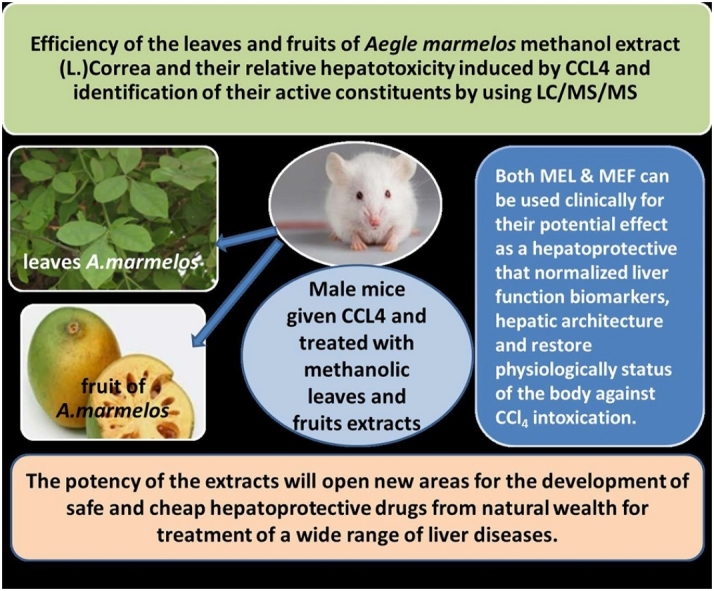

## 1 Introduction

Carbon tetrachloride is one of the xenobiotics that have been reported to induce acute and chronic tissue injuries and is a well-established hepatotoxic. CCl_4_ was reported to increase serum hepatotoxicity and nephrotoxicity markers. Free radicals play an important role in CCl_4_-induced liver and renal damage process [1]. Histopathologically exposure to CCl_4_ caused hepatic steatosis, centrilobular necrosis, and cirrhosis in the liver and acute tubular necrosis in the kidney [2].

*Aegle marmelos* (L.) Correa is a tree belongs to family Rutaceae found all over India and commonly known as the bale fruit tree. It is used in traditional medicine for the treatment of various diseases such as dysentery, fever, diabetes, asthma, heart problems, ophthalmic, hemorrhoids and urinary problems in humans [3]. Fruit of *A. marmelos* is prescribed in the treatment of tuberculosis, hepatitis and used against stomach complications [4].

The aqueous decoction of the leaves has been reported to have a significant hypoglycemic effect and helps in regeneration of a damaged pancreas [5]. Also, extract of the leaves has been reported to have chemopreventive potential especially against chemical carcinogenesis [6]. Aqueous and alcoholic extracts of *A. marmelo*s leaves reported to have cardiotonic effect. The major constituents of leaves extract were tannins, skimmianin, essential oil, sterol, triterpenoids, flavonoids and coumarins [7].

The chemical constituents of the essential oil of *A. marmelos* which recorded promising antifungal and antimicrobial activities also, the hypoglycemic effect of methanol extract and mucilage of *A. marmelos* fruits were recently reported by [8,9]. In addition, two new cytotoxic alkaloids of the furoquinoline-type were isolated from *A. marmelos* leaves [10].

Based on the broad range of activities known for both leaves and fruits of *A. marmelo*s, the present research designed to evaluate the efficiency of the of the plant in ameliorating hepatotoxicity induced by CCl_4_. This was accompanied by identification without isolation for the major active constituents by using liquid chromatography mass spectrometry (LC/MS/MS) technique, for the first time.

## 2 Materials and methods

### 2.1 Plant material

The fresh leaves and fruits of *A. marmelos* (L.) were collected from in El-Zohrya botanical garden, Giza, Egypt in April 2008. The plant was identified by Mrs. Therese Labib, consultant of taxonomy at the ministry of agriculture and the former director of El-Orman Botanical Garden. A voucher specimen (No. 00017 1Ac 04-02-05-17) was kept at the Herbarium of El-Orman Botanical Garden.

### 2.2 Method of extraction

The collected leaves and fruits were air dried, the powdered plant was kept in tightly-closed containers. Leaves and fruits (1 kg of each part) were successively extracted using a soxhlet apparatus with solvents of increasing polarities (pet. ether, chloroform, ethyl acetate and methanol). The MEL & MEF were evaporated to dryness at 40 °C to yield 10.9, 12.0% w/w respectively, the chemical constituents of leaves and fruits were determined by LC/MS/MS technique as revealed in Table 1. The hepatoprotective effect against CCl_4_ toxicology was also performed.

**Table 1:** LC/MS/MS of Methanol Extract leaves and fruit of *A. marmelos* (L.) Correa.

Identified Compounds	M.F.	Leaves	Fruits	R*_t_*	B.P	[M+H]^+^
Rutin	C_27_H_30_O_16_	+	–	14.27	303	611
Aeglemarmelosine	C_16_H_15_NO_2_	+	–	15.24	214	255
Kaempferol-3-*O*-rutinoside	C_27_H_30_O_15_	+	–	16.40	287	595
Marmesiline	C_22_H_25_NO_4_	+	+	17.21	368	368
Aegelinosides	C_24_H_29_NO_8_	+	–	17.99	280	460
Shahidine	C_18_H_17_NO_2_	+	+	22.06	214	280
*N*-2-Methoxy-2-(4-methoxyphenyl) Ethylcinnamide	C_19_H_21_NO_3_	+	–	22.09	280	312
Anhydromarmeline	C_22_H_23_NO_2_	+	–	26.29	334	334

R*_t_*: retention time; B.P: bas peak; M.F.: molecular formula.

### 2.3 LC/MS/MS condition

The high-resolution mass spectra were obtained with an LTQ-Orbitrap spectrometer (ThermoFisher, USA) equipped with a HESI-II source. The spectrometer was operated in positive mode (1 spectrums^−1^; mass range: 200–1000) with nominal mass resolving power of 60,000 at *m/z* 400 with a scan rate of 1 Hz with automatic gain control to provide high-accuracy mass measurements within 2 ppm deviation using an internal standard; bis(2-ethylhexyl)phthalate: *m/z* = 391.284286. The spectrometer was attached with an Agilent 1200 HPLC system (Santa Clara, USA) consisting of LC-pump, PDA detector (λ = 205 nm), auto sampler (injection volume 10 μL) and column oven (30 °C). MS/MS experiments were performed by CID (Collision Induced Decay, 35 eV) mode. Following parameters were used for experiments: spray voltage 5 kV, capillary temperature 260 °C, tube lens 70 V. Nitrogen was used as sheath gas (50 arbitrary units) and auxiliary gas (five arbitrary units). Helium served as the collision gas. The separations and purifications were performed by using a Nucleodur Gravity column (50 × 2 mm, 1.8 μm particle size) from Macherey–Nagel (Düren, Germany) with a H_2_O (+0.1% HCOOH, + 10 mM NH_4_Ac) (A)/acetonitrile (+0.1% HCOOH) (B) gradient (flow rate 300 μL min^−1^). Samples were analyzed by using a gradient program as follows: 90% A isocratic for 2 min, linear gradient to 100% B over 13 min, after 100% B isocratic for 5 min, the system returned to its initial condition (90% A) within 0.5 min, and was equilibrated for 4.5 min.

### 2.4 Chemicals

All the reagents used were of analytical grade and were obtained from Sigma (USA), Randox (U.K), Biodiagnostic and Stanbio (Egypt) Chemicals Co.

### 2.5 Animals

The experimental animals were intact male mice (strain CDI) obtained from animal house of Theodor Bilharz Institute, Cairo, Egypt ranges in weight from 30 to 35 g, they were fed on standard protein rich pellet diet (*ad- libitum*) obtained from EL-Kahira company for Oil and Soap. Anesthetic procedures complied with legal ethical guidelines approved by the Ethical Committee of the National Research Centre (Egypt) with approval No (13-018) for hepatoprotective study. Adequate measures were taken to minimize animal pain and discomfort. Also, the experiments were carried out in accordance to NIH guidelines for care and use of animals

### 2.6 Experimental design

Sixty mice were divided into six groups of 10 mice each asfollows:

G1 Control group, received orally saline (0.2 ml/100 g) for 30 days.

G2 *A. marmelos* leaves group, where mice received orally 250 mg/kg body weight/day methanol extract of *A. marmelos* leaves for 30 successive days [11].

G3 *A. marmelos* fruits group, where mice received orally 125 mg/kg body weight/day methanol extract of *A. marmelos* fruits for 30 successive days [12].

G4 CCl_4_-intoxicated group, where mice injected intraperitoneal (i.p.) with fresh mixtures of equal volumes of CCl_4_ and olive oil for two consecutive days at the dose 0.1 ml/100 g body weight/day [4].

G5 *A. marmelos* leaves treated group, where intoxicated mice treated orally with A*. marmelos* leaves extract (250 mg /kg) body weight/day at the 3rd day post the last dose of CCl_4_ and for 30 successive days.

G6 *A. marmelos* fruits treated group where intoxicated mice were treated orally with *A. marmelos* leaves extract (125 mg /kg) body weight/day at the 3rd day post the last dose of CCl_4_ and for 30 successive days.

### 2.7 Preparation of samples

At the end of the experiment, mice were fasted overnight for more than 12 h, and then blood sample was withdrawn and allowed to clot, then centrifuged, serum stored at −80 °C to be used for biochemical analysis. Mice were then sacrificed and 0.5 g liver tissue was homogenized in 4.5 ml saline solution (0.9%) to yield 10% liver homogenate freshly used or kept frozen at −80 °C for biochemical analysis.

### 2.8 Histopathology

Liver specimens were fixed in 10% formalin, sectioned (4 μm thick) and stained with Hematoxyline and Eosin stain. They were examined using light microscopy [13].

### 2.9 Calculation

Percentages of change and improvement were calculated according to the following equations [14].

$$\%\;\text{ Change}=\frac{\text{Mean}\;\text{of}\;\text{treated}\;\;-\;\;\text{Mean}\;\text{of}\;\text{control}}{\text{Mean}\;\text{of}\;\text{control}}\times 100$$

$$\%\;\text{Improvement}=\frac{\text{Mean}\;\text{of}\;\text{disease}\;\;-\;\;\text{Mean}\;\text{of}\;\text{treated}}{\text{Mean}\;\text{of}\;\text{control}}\times 100$$

### 2.10 Statistical analysis

Statistical analysis was carried out by one way analysis of variance (ANOVA), the SPSS computer program (Version 11), combined with post-Hoc (LSD; Least Significance Difference where significance is considered at P ≤ 0.05).

### 2.11 A. Determination of serum biochemical parameters

Total cholesterol (TC) level [15], high and low density lipoprotein – cholesterol (HDL-c and LDL-c) [16], triglyceride [17], total lipids [18] and phospholipids [19] were determined.

### 2.12 B. Determination of glycolytic enzyme activities in liver tissue homogenate

Estimation of succinate dehydrogenase enzyme activity (EC 1.3.99.1) [20], lactate dehydrogenase activity (EC 1.1.1.27) [21], total bilirubin [22], alanine and aspartate aminotransferases enzyme activities (ALT & AST) [23,24], alkaline phosphatase [25],

total protein content [26] and serum albumin [27] were also measured.

## 3 Result

### 3.1 The methanol extracts of leaves and fruits of *A. marmelos* were analyzed by LC/MS/MS using computer program X-Caliber

Six alkaloidal compounds namely aeglemarmelosine, marmesiline, aegelinoside, shahidine, *N*-2-methoxy-2-(4-methoxyphenyl) ethylcinnamide and anhydromarmeline had been identified and two flavonoids, rutin and kaempferol-3-*O*-rutinoside in the methanol extracts of leaves. Additionally, two alkaloids, marmesiline and shahidine in the methanol extracts of fruits had been identified. The identification of the compounds was based on the comparison of their mass spectral data and the fragmentation pattern including molecular ion peak and their CID fragmentations pattern with the previously reported data as shown in Table 1.

### 3.2 Lipid profile in normal, CCl_4_ intoxicated and treated mice

**Fig. 1:**
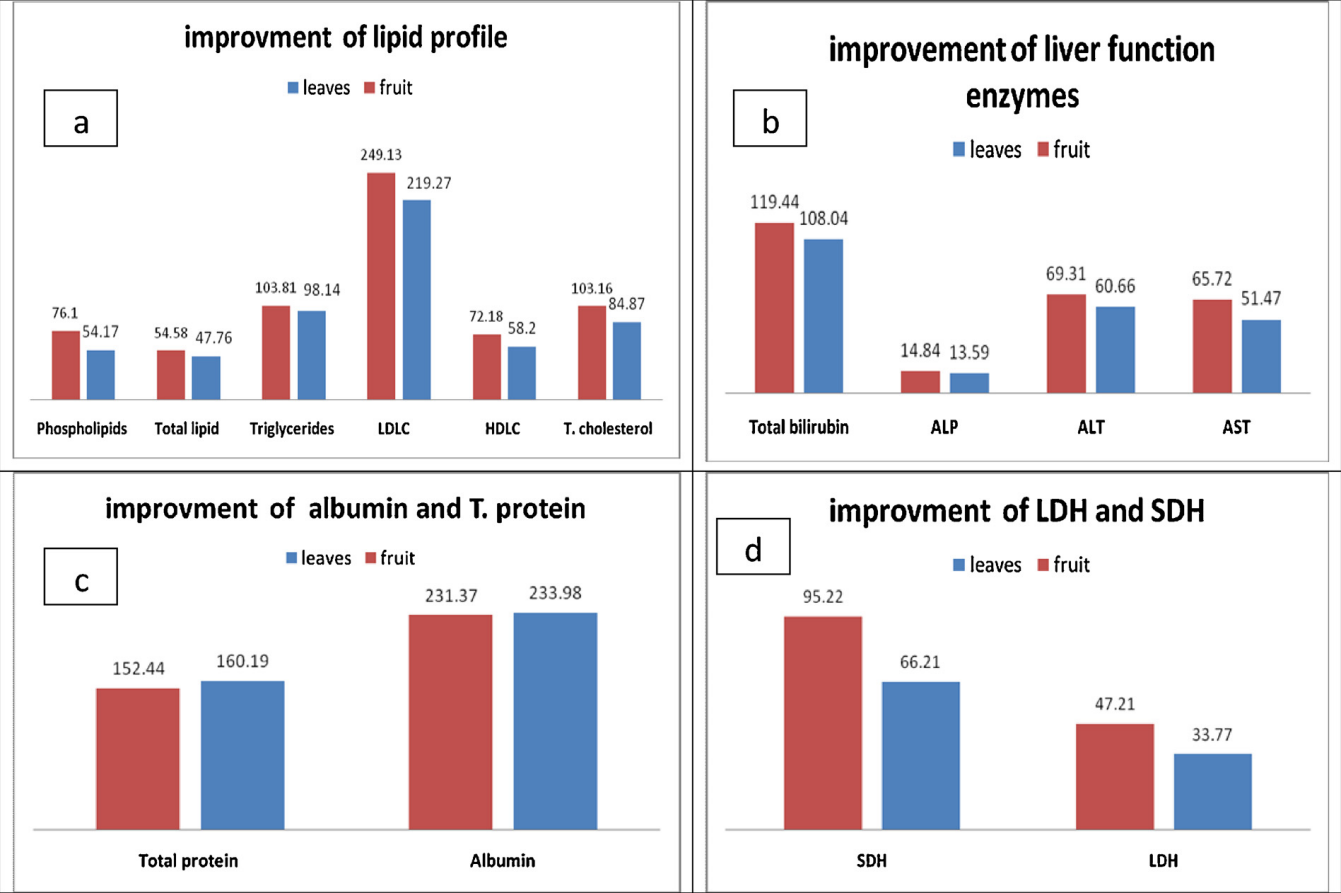
illustrating percentages of improvement in different biomarkers of CCl4-intoxicated rats treated with leaves and fruits of *A. marmelose*.

Table 2 revealed that significant reduction in total cholesterol, triglycerides and total lipid in normal mice treated with MEL & MEF. While, the study did not observe significant changes in HDL-C, LDL-C and phospholipids levels in control mice treated with MEL & MEF compared to untreated control mice. On the other hand, significant increase in total cholesterol, LDL-C, triglycerides, total lipid and phospholipids in CCl_4_ intoxicated mice with percentages of increase reached to 127.38, 265.25, 89.49, 74.23 and 86.89%, respectively compared to normal control. However, HDL-C exhibited significant decrease as compared to normal control mice (35%). The CCl_4_ intoxicated mice treated with MEL & MEF, marked improvement noticed in total cholesterol with percentage of improvement recorded 84.87 and 103.16%, respectively. While HDL-C level showed improvement with percentage amounted 72.18 and 58.20%, respectively. Also, amelioration was noticed in LDL, triglycerides, total lipid and phospholipids with percentage 219.27, 98.14, 47.76 and 54.17%, respectively, for methanol leaves and 249.13, 103.81, 54.58 and 76.10%, respectively for methanol fruits as in (Fig. 1-A).

**Table 2:** Lipid profile in normal, CCl4 intoxicated and treated mice.

Parameters Groups	normal control (1)	Leaves- treated normal (2)	Fruit- treated normal (3)	CCl_4-_injected mice (4)	Leaves-treatrdCCl_4_ intoxicated mice (5)	Fruit- treatrdCCl_4_ intoxicate d mice (6)	LSD
T. cholesterol	58.83 ± 1.46 (2-6)	53.88 ± 2.54 (1,4,5,6)	52.30 ± 1.60 (1,4–6)	133.77 ± 3.26 (1,2,3,5,6)	83.84 ± 3.48 (1–4,6)	73.08 ± 2.49 (1,2–,5)	0.000
HDL- - Cholesterol	25.60 ± 0.65 (4,5,6)	26.08 ± 0.45 (4,5,6)	26.06 ± 0.70 (4,5,6)	16.51 ± 1.76 (1,2,3,5,6)	31.41 ± 1.72 (1–4)	34.99 ± 1.69 (1–4)	0.000
LDL- cholesterol	17.18 ± 1.81 (4–6)	14.93 ± 0.76 (4–6)	14.75 ± 0.69 (4–6)	62.75 ± 3.42 (1,2,3,5,6)	25.08 ± 1.32 (1,2,3,4,6)	19.95 ± 0.89 (1–5)	0.001
Triglycerides	85.33 ± 2.49 (2–6)	76.88 ± 1.69 (1,4,6)	74.83 ± 1.24 (1,4)	161.69 ± 3.18 (1,2,3,5,6)	77.95 ± 1.89 (1,4,6)	73.11 ± 2.17 (1,2,4,5)	0.000
Total lipid	990 ± 7.89 (1,3–6)	820 ± 38.6 (1,3–,6)	736 ± 27.17 (1,2,4–6)	1725 ± 34.67 (1,2,3,5,6)	1252 ± 41.1 (1–4,6)	1185. ± 23.80 (1–,5)	0.000
Phospholipids	86.37 ± 3.75 (4,5)	82.51 ± 7.54 (4,5)	78.19 ± 6.59 (4–6)	161.42 ± 10.16 (1,2,3,5,6)	114.63 ± 19.37 (1–4,6)	95.71 ± 14.43 (3–5)	0.000

Data are mean (±S.D) of six mice in each group and are express in mg/dl.

Statistics is carried out by one – way ANOVA (SPSS computer program and the analysis of variance is carried out.

By post-hoc and LSD (least significant difference), where the mean difference is significant at P ≤ 0.05 level.

### 3.3 Liver function enzyme activities in normal, CCl_4_-intoxicated and treated mice

Table 3 showed insignificant changes in all liver function enzyme activities and total bilirubin in normal mice treated with MEL & MEF compared to normal untreated one. While, significant increase in AST, ALT, ALP enzyme activities and total bilirubin level in CCl_4_-intoxicated mice with percentage increase 80.31, 124.54, 22.81 and 155.54%, respectively. Remarkable improvement was noticed in liver function biomarkers of intoxicated mice treated with both extracts exhibited percentages of improvement 51.47, 60.66, 13.59 and 108.04%, for AST, ALT, ALP and total bilirubin level for leaves extract. While, the percentages of amelioration reached at 65.72, 69.31, 14.84 and 119.44%, respectively for fruits treated one. So, the higher ameliorative effect for fruits extract followed by leaves (Fig. 1-B).

**Table 3:** Liver function enzyme activities in normal, CCl4- intoxicated and treated mice.

Parameters Groups	Normal control (1)	Leaves -treated normal (2)	Fruit -treated normal (3)	CCl_4-_ injected mice (4)	leaves treatrdCCl_4_ intoxicated mice (5)	Fruit- treatrdCCl_4_ intoxicated mice (6)	LSD
AST (U/L)	49.10 ± 0.64 (4–6)	48.78 ± 0.49 (4–6)	49.17 ± 0.67 (4–,6)	88.53 ± 1.61 (1,2,3,5,6)	63.26 ± 2.94 (1,2,3,5.6)	56.26 ± 1.1 (1–,5)	0.000
ALT (U/L)	26.69 ± 1.14 (4–6)	26.76 ± 0.36 (4–6)	27.6 ± 0.64 (4–6)	59.93 ± 0.43 (1,2,3,5,6)	43.74 ± 2.44 (1,2,3,5.6)	41.43 ± 2.21 (1–5)	0.000
ALP (U/L)	179.17 ± 2.90 (4–,6)	180.99 ± 0.83 (4–6)	182.43 ± 1.92 (4–6)	220.04 ± 8.19 (1,2,3,5,6)	195.65 ± 3.67 (1,2,3,5.6)	193.45 ± 4.02 (1–5)	0.002
Total bilirubin (mg/dl)	51.75 ± 0.53 (4–6)	50.64 ± 1.64 (4–6)	50.36 ± 1.28 (4–6)	132.24 ± 2.40 (1,2,3,5,6)	76.33 ± 4.2 (1,2,3,5.6)	70.43 ± 0.52 (1–5)	0.001

Data are mean (±S.D) of six mice in each group and all enzymes are express in U/L. while total bilirubin is expressed in mg/dL.

Statistics is carried out by one – way ANOVA (SPSS computer program and the analysis of variance is carried out by post-hoc and LSD (least significant difference), where the mean difference is significant at P ≤ 0.05 level.

### 3.4 Albumin level and total protein content in normal, CCl_4_-intoxicated and treated mice

Table 4 revealed insignificant increase in albumin level and total protein content in normal MEL & MEF treated mice as compared to control group. While, CCl_4_ intoxicated mice exhibited a significant increase in both albumin and total protein levels with percentages amounted 287.58 and 146.57%, respectively as compared to normal control. Marked improvement was observed in the albumin level post treatment of intoxicated mice with MEL & MEF, with improvement percentages 233.98 and 231.37%,respectively. However, total protein content showed insignificant change post treatment of intoxicated mice with both leaves and fruits as compared to normal mice (Fig. 1-C).

**Table 4:** Albumin level and total protein content in normal, CCl4 intoxicated and treated mice.

Parameters Groups	Normal control (1)	Leaves -treated normal (2)	Fruit- treated normal (3)	CCl4- injected mice (4)	Leaves- treatrdCCl_4_ intoxicated mice (5)	Fruit- treatrdCCl_4_ intoxicate d mice (6)	LSD
Albumin	1.53 ± 0.02 (4,5)	1.57 ± 0.11 (4,5)	1.94 ± 0.04 (4-6)	5.93 ± 0.25 (1,2,3,5,6)	2.35 ± 0.39 (1–4,6)	2.39 ± 0.40 (1–5)	0.000
Total protein	111.93 ± 2.02 (4)	121.58 ± 1.77 (4)	124.33 ± 3.72 (4)	275.99 ± 4.78 (1,2,3,5,6)	96.68 ± 3.41 (4)	105.36 ± 3.20 (4)	0.002

Data are mean (±S.D) of ten mice in each group and albumin is expressed in mg/dL, while total protein content in g/L.

Statistics is carried out by one – way ANOVA (SPSS computer program and the analysis of variance is carried out by post-hoc and LSD (least significant difference), where the mean difference is significant at P ≤ 0.05 level.

### 3.5 LDH and SDH enzyme activities in normal, CCl_4_-intoxicated and treated mice

Table 5 declared insignificant change in LDH and SDH enzyme activities in mice treated with MEL & MEF as compared to untreated normal control mice. A significant increase was noticed in LDH and SDH enzyme activities in CCl_4-_intoxicated mice with percentages increase amounted 68.95 and 155.63%, respectively as compared to normal control group. Moreover, the percentages of amelioration of LDH and SDH activities recorded 33.77, 66.21%, for methanol leaves, 47.21 and 95.22%, for methanol fruits extracts, respectively as in (Fig. 1D).

**Table 5:** Levels of LDH and SDH enzyme activities in normal, CCl_4_- intoxicated and treated mice.

Parameters Groups	Normal control (1)	Leaves treated normal (2)	Fruit treated normal (3)	CCl_4_ treated mice (4)	leaves treatrdCcl_4_ intoxicated mice (5)	fruit treatrdCcl_4_ intoxicate d mic (6)	LSD
LDH (u/ml)	78.560 ± 3.13 (4–6)	77.93 ± 2.55 (4–,6)	80.83 ± 1.58 (4–,6)	132.73 ± 2.08 (1,2,3,5,6)	106.32 ± 4.24 (1–4,6)	95.61 ± 4.71	0.000
SDH (u/ml)	0.293 ± 0.01 (4–6)	0.300 ± 0.02 (4–,6)	0.287 ± 0.02 (4-6)	0.749 ± 0.07 (1,2,3,5,6)	0.555 ± 0.02 (1–4,6)	0.47 ± 0.03	0.004

Data are mean (±S.D) of six mice in each group and all enzymes are express in U/ml. while total bilirubin is expressed in mg/dl.

Statistics is carried out by one – way ANOVA (SPSS computer program and the analysis of variance is carried out by post-hoc and LSD (least significant difference), where the mean difference is significant at P ≤ 0.05 level.

### 3.6 Histopathological investigation

**Fig. 2:**
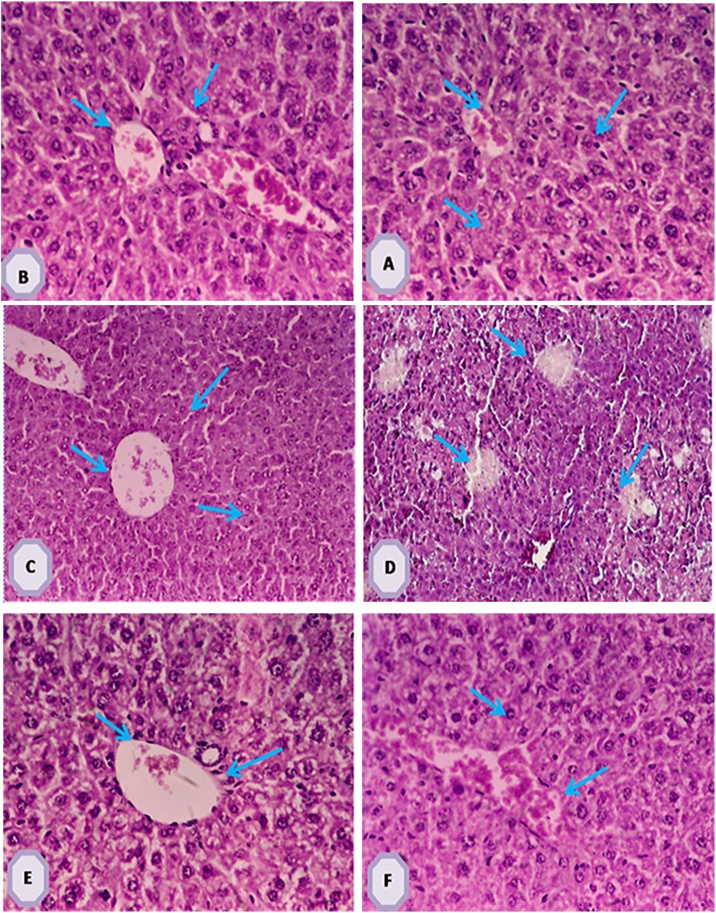
**(A)** Photomicrograph of control mice with preserved hepatic architecture 200× (H and E stain). **(B)** Photomicrograph of leaves treated control mice with normal hepatic cell 200× (H and E stain). **(C):** Photomicrograph of fruits treated control mice with no change in hepatic architecture 100× (H and E stain). **(D)** Photomicrograph of section through the liver CCl_4_-group showing lymphocyte infiltration and dilated as well as congested veins 100× (H and E stain). **(E)** Photomicrograph (E) of leaves-treated CCl_4_ mice showing the central vein (CV) and hepatocytes, the liver shows normal histological profile 200× (H & E stain).**(F)** Photomicrograph (F) of fruits-treated CCl_4_ mice show normal hepatocytes 200× (H and E stain).

**Fig. 3:**
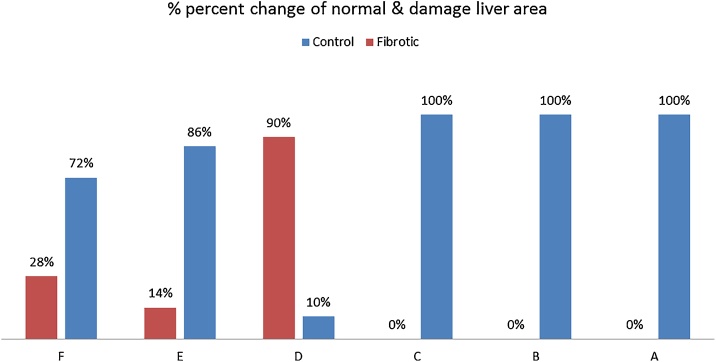
Illustrate % of normal & damage liver area.

Histopathological sections of mice liver tissues (H & E 200×) Fig. 2(A): control section of mice showing normal hepatic cells. Fig. 2(B&C): sections of control mice given methanol extract of leaves (200×) & extract fruits (100×) respectively showing no change of hepatocyte closed to control hepatic cells. (D): CCl_4_ induced hepatotoxic section of mice showing hepatocellular degeneration, cytoplasmic vacuolization and necrosis of hepatocytes and partial infiltration with inflammatory cells.(E&F): sections of CCl_4_ injected mice and treated with MEL & MEF (200×) respectively treated revealed a significant enhancement in hepatic cells with normal sinusoid nearly normal parenchyma architecture, in addition Fig. 3 illustrate % percent change of normal & damage liver area.

## 4 Discussion

*A. marmelos* is a subtropical plant. The compounds identified by LC/MS/MS either of alkaloidal nature or flavonoidal nature were in agreement with the previously reported literature, where *aeglemarmelosine* was isolated previously [8,28], from the root *A. marmelos* using flash column chromatography *aeglemarmelosine* considered as an oxazoline derivative with [M] ^+^ 254 Table 1.

Marmesiline was isolated with [M]^+^367 from the acetone extract of the fruits using flash column chromatography [29].

It was reported that series of phenylethylcinnamides alkaloids were isolated included aegelinoside with [M]^+^ 459 from the alcoholic extract of *A. marmelos* leaves and also anhydromarmeline with [M]^+^ 333 and their structures were identified by different spectroscopic tools [30]. Shahidine isolated from the methanol extract of leaves which considered as the parent compound of aegline with oxazoline ring, with [M] ^+^ 279 [31].

*N*-2-Methoxy-2-(4-methoxyphenyl)ethylcinnamide isolated from methanol extract of leaves of *A. marmelos* with [M]^+^ 311 [32]. Mass spectral fragmentations of a number of *O*-diglycosyl flavonoids, including rutin and kaempferol diglycosides using ESI/MS, which confirmed the structure as well as the glycosidic inter-linkage [33].

All findings here reported for the first time the isolation of aeglemarmelosine and marmesiline from the leaves, besides, shahidine was isolated from the fruits of *A. marmelos*.

The present results showed significant decrease in triglycerides, total lipid and total cholesterol, while significant increase in albumin and total protein content in normal animal treated with leaves and fruits extracts of *A. marmelos*, while insignificant changes in other parameters. These significant effects might be due to effective quenching of free radicals cause decrease in hepatic lipid peroxide. Moreover *A. marmelos* is considered to be a natural antioxidant [34].

CCl_4_-intoxication is associated with reduction in phospholipids content that is essential for membrane structure and function [35]. The increase in serum phospholipids in mice injected with CCl_4_ may be due to increased peroxidation of membrane phospholipids, releases free fatty acids via phospholipase A2 and hyper triglyceridemia observed in CCl_4_, intoxication may be attributed to inhibition in the activity of lipoprotein lipase in the heart, resulting in decreased uptake of triglycerides from the circulation [36].

The results of this study revealed that a regular administration of *A. marmelos* leaves and fruits extracts (250 mg/kg and 125 mg/kg) respectively for 30 successive days nearly normalized lipid profile in CCl_4_-intoxicated mice and not only lowered total cholesterol, triglycerides, and LDL but also improved the HDL. CCl_4_-intoxicated mice showed an increase in the activities of serum ALT and AST. The increase in these enzyme activities agreed with the previous studies on the hepatotoxic effect of CCl_4_ on liver function [37]. CCl_4_ treatment caused significant decrease in cell viability and the toxin treatment initiated lipid peroxidation (LPO), promoted leakage of enzymes like AST and ALT [38]. Regarding serum ALP enzyme activity, significant increase in its activity in CCl_4_-intoxicated mice was recorded as compared to control group [39].

CCl_4_-intoxicated mice showed an increase in serum total bilirubin level as compared to control. Administration of both leaves and fruits extracts (250 mg and 125 mg/kg body weight/day) respectively, for 30 successive days revealed their ability to restore the normal status of the intoxicated liver.

The present results demonstrated significant increase in total protein content and albumin level after CCl_4_-intoxication. The ameliorating effect may be related to *A. marmelos* leaves or fruits extracts exhibited autolysis and preserved the architecture of cell membrane that prevent leakage of cell enzymes and constituent [40].

The present research demonstrated significant increase in the activities of LDH and SDH glycolytic enzymes in liver tissue of CCl_4_-intoxicated mice and marked improvement after treatment with both extracts of *A. marmelos*.

This may be attributed to the different biologically active constituents which were identified by LC/MS/MS. Briefly, rutin reported to have hepatoprotective activity as it prevented CCl_4_-induced elevation in serum ALT and AST enzyme activities [41]. In addition, kaempferol 3-*O*-rutinoside has protective effects against acute CCl_4_-induced oxidative liver damage [42]. Moreover, the observed activity for the fruits extract may be due to their contents of mucilage [9], which may enhance liver toxicity by capture trichloromethyl radical (^•^CCl_3_) that is responsible for the hepatic injury of the CCl_4_ [43].

Fibrosis of liver, which is a mark of chronic liver disease, characterized by hepatic excessive accumulation of connective tissue as an indicator of progressive hepatic injury [44]. Histopathological examinations of intoxicated liver also supported the biochemical results, by CCl_4_-exposed mice for 48 h showed vacuolar degeneration and micro-fatty changes. These results were in agreement with [45,46], who reported that after CCl_4_ treatment, significant liver damage was observed with classic histology of cirrhosis, coagulated necrosis, massive fibrosis, fatty degeneration and formation of regenerative nodules, the treatment of methanolic extracts of *Juniperus phoenicea* and *Cupressus sempervirens* for 30 successive days showed amelioration effect on CCl_4_ induced hepatotoxicity, decreased steatosis, reduction in central vein dilation and normalization of hepatocytes [46].

From the above mentioned results, it was concluded that rutin and quercetin are natural polyphenolic flavonoids which reported to show a wide range of biological and health-promoting effects such as antioxidant, anticancer, hepatoprotective, antidiabetic and anti-inflammatory [47,48]. In the present study the methanol extracts of *Aegle* leaves and fruits contained many different biologically active compounds such as flavonoids and alkaloids, which potentiate each other to produce synergistic effect and prevention of the hepatic stress. Accordingly, the methanol extract of the plant under investigation recommended to use as an alternative natural product to produce hepatoprotective and hypolipidemic agents, also they could be used as antioxidant agents for controlling free radicals in diabetes.

## 5 Conclusion

It could be concluded that, control mice treated with *A. marmelos* leaves and fruits methanol extracts exhibited significant reduction in total lipid, total cholesterol and triglycerides as compared to control group. CCl_4_-intoxicated mice exhibited significant increase in lipid profile, liver function and glycolytic enzymes in addition to albumin level and total protein content. Marked amelioration was noticed in the levels of all the measured biochemical parameters in CCl_4_-intoxicated mice as a result of treatment with MEL & MEF with more marked effect for fruits than leaves that in turn helping in persevered and normalized liver architecture, can be due to their contents of mucilage. Hence, both leaves and fruits extracts can be applied clinically suggesting their potential use as a hepatoprotective and effective agent that normalized liver functions and restore physiologically status of the body especially against CCl_4_ intoxication.

The potency of the extracts will open new areas for the development of safe and cheap hepatoprotective drugs from natural wealth for treatment of a wide range of liver diseases.

## 6 Authors’ contributions

All authors carried out the experiments, drafted the manuscript and Participated in the design of the study. Nabaweya A. Ibrahim, Magdy M. D. Mohammed and Doaa Abd Al-Hady are responsible for Plant material and their extractions. Hanan F. Aly, Sanaa A. Ali are responsible for the biochemical and histological studies. All authors have read and approved the final manuscript.

## Conflict of interest

The authors have no financial or personal conflicts of interest to declare in relation to this article.

Transparency Document
